# Developing Equations for Converting Digestible Energy to Metabolizable Energy for Korean Hanwoo Beef Cattle

**DOI:** 10.3390/ani11061696

**Published:** 2021-06-07

**Authors:** Ridha Ibidhi, Rajaraman Bharanidharan, Jong-Geun Kim, Woo-Hyeong Hong, In-Sik Nam, Youl-Chang Baek, Tae-Hoon Kim, Kyoung-Hoon Kim

**Affiliations:** 1 Department of Eco-Friendly Livestock Science, Institutes of Green Bio Science and Technology, Seoul National University, Pyeongchang 25354, Gangwon-do, Korea; ridha@snu.ac.kr (R.I.); forage@snu.ac.kr (J.-G.K.); 2 Department of Agricultural Biotechnology, College of Agriculture and Life Sciences, Seoul National University, Seoul 08826, Korea; bharanidharan7@snu.ac.kr; 3 Department of International Agricultural Technology, Graduate School of International Agricultural Technology, Seoul National University, Pyeongchang 25354, Gangwon-do, Korea; hwh1195@snu.ac.kr (W.-H.H.); whyhoon1@snu.ac.kr (T.-H.K.); 4 Division of Applied Bio-Industry Science, School of Animal Life Convergence Science, Hankyoung National University, Anseong-si 17579, Gyeonggi-do, Korea; isnam@hknu.ac.kr; 5 Animal Nutritional and Physiology Team, National Institute of Animal Sciences, Rural Development Administration, Wanju 55365, Jeollabuk-do, Korea; chang4747@korea.kr

**Keywords:** beef cattle, digestible energy, metabolizable energy, mixed model

## Abstract

**Simple Summary:**

The available energy in feedstuff represents the largest proportion of the total cost for intensive beef production. Therefore, the energy content of feeds must be known before diet formulation. The determination of digestible energy (DE) and metabolizable energy (ME) values by animal experiments is both time-consuming and costly. Predictive equations to estimate the ME from DE can be useful for feed ingredient evaluations and diet formulations. A range of regression equations were developed in the present study, taking into consideration the gender and body weights of the animals, as well as the feed nutrients, to predict the relationship between the DE and ME. An evaluation of these equations suggested predicting the ME value based on ME = 0.9215 × DE − 0.1434 (R^2^ = 0.999). The generation of these predictive equations represents a step towards updating the ME:DE default conversion factor value of 0.82 adopted from the National Research Council to meet the ME requirements of beef cattle in Korea. The new recommended predictive equation enables the adjustment of the nutrient requirements, thus enhancing animal productivity and maximising the economic return for beef farmers.

**Abstract:**

This study was performed to update and generate prediction equations for converting digestible energy (DE) to metabolizable energy (ME) for Korean Hanwoo beef cattle, taking into consideration the gender (male and female) and body weights (BW above and below 350 kg) of the animals. The data consisted of 141 measurements from respiratory chambers with a wide range of diets and energy intake levels. A simple linear regression of the overall unadjusted data suggested a strong relationship between the DE and ME (Mcal/kg DM): ME = 0.8722 × DE + 0.0016 (coefficient of determination (R^2^) = 0.946, root mean square error (RMSE) = 0.107, *p* < 0.001 for intercept and slope). Mixed-model regression analyses to adjust for the effects of the experiment from which the data were obtained similarly showed a strong linear relationship between the DE and ME (Mcal/kg of DM): ME = 0.9215 × DE − 0.1434 (R^2^ = 0.999, RMSE = 0.004, *p* < 0.001 for the intercept and slope). The DE was strongly related to the ME for both genders: ME = 0.8621 × DE + 0.0808 (R^2^ = 0.9600, RMSE = 0.083, *p* < 0.001 for the intercept and slope) and ME = 0.7785 × DE + 0.1546 (R^2^ = 0.971, RMSE = 0.070, *p* < 0.001 for the intercept and slope) for male and female Hanwoo cattle, respectively. By BW, the simple linear regression similarly showed a strong relationship between the DE and ME for Hanwoo above and below 350 kg BW: ME = 0.9833 × DE − 0.2760 (R^2^ = 0.991, RMSE = 0.055, *p* < 0.001 for the intercept and slope) and ME = 0.72975 × DE + 0.38744 (R^2^ = 0.913, RMSE = 0.100, *p* < 0.001 for the intercept and slope), respectively. A multiple regression using the DE and dietary factors as independent variables did not improve the accuracy of the ME prediction (ME = 1.149 × DE − 0.045 × crude protein + 0.011 × neutral detergent fibre − 0.027 × acid detergent fibre + 0.683).

## 1. Introduction

Energy is a vital component for biological reactions and an important nutrient to meet the requirements for the maintenance, growth and reproduction of beef cattle. The energy requirements depend mainly on age, gender, body weight (BW), animal genotype, physiological state and environment [1]. To meet their energy requirements, beef cattle rely on an intake of energetic feeds. Beef cattle diets that do not meet their energy requirements may result in a failure to obtain the expected performances of the animals. Therefore, the available energy of feedstuffs is an important component of nutritional programs in beef cattle operations.

Hanwoo is a cattle breed native to the Republic of Korea and is one of the most economically important domestic animals in the country. The Hanwoo beef industry has experienced a considerable change between 2005 and 2017, with the total number of cattle increasing from 1.8 to 2.6 million head and an improvement of Hanwoo cattle performance in terms of the quantity and quality. The average carcass weight of Hanwoo steers increased from 343 to 437 kg and the marbling score improved from 3.6 to 5.6 within the same period, andthis trend affected the basal metabolism of the animals. Hence, Hanwoo beef cattle tended to have higher metabolic rates and require more energy for maintenance and production. Currently, farmers rely more on the use of high energy-based concentrate diets imported mainly from international markets [2]. Therefore, the accurate prediction of energy concentration of feed ingredients is a key driver for diet formulation to optimise the production costs. The evaluation of the energy content of Hanwoo beef cattle feeds is usually based on their digestible energy (DE) or metabolizable energy (ME) contents in the Korean Feeding Standards for Hanwoo [3]. The ME is calculated from the DE using a constant factor of 0.82, which was suggested by the National Research Council (NRC) [4,5,6]. However, several studies have reported that a fixed ME:DE is an oversimplification and does not represent the diversity of existing feedstuffs [7,8,9]. Vermorel and Bickel [9] reported considerable variances in the ME:DE with a clear pattern, e.g., when the diet has higher gross energy contents, the ratio is greater (up to 94%). More recently, Galyean et al. [8] performed meta-regression linear analyses to update the ME and DE relationship (ME = 0.9611 × DE − 0.2999) based on 23 respiration calorimetry studies (*n* = 87 means) from different cattle breeds. Scarce calorimetric studies have been conducted in Korea to evaluate the efficiency of converting the DE to ME of the Korean native beef cattle (Hanwoo) [10]. Kim et al. [10] estimated the ME:DE of Hanwoo steers weighing 376.6 and fed rice straw (44%) and concentrate (56%) at three different energy levels (0.8, 1.2 and 1.6 times the maintenance), and the ME:DE ranged between 83% and 90%. The above results imply that the NRC equation for converting the DE to ME is not adequate for Korean Hanwoo Beef Cattle, since the Hanwoo breed is apparently more efficient in transforming the DE into ME. On the other hand, the ME:DE fixed conversion factor recommended by the NRC [4,5,6] was developed using old data obtained over 40 years ago and did not cover the specificity of many breeds and the regional feed quality. Galyean et al. [8] reported different ME:DE conversion factors according to the breed that ranged from 0.69 in Brahman heifers to 0.96 in Jersey steers.

Therefore, we hypothesised that Hanwoo beef cattle have been fed more digestible diets with different ME:DE conversion factors compared to the NRC conversion factor. Therefore, there is an urgent need to update this conversion factor according to the Korean context (diet and genotype). Hence, the aim of this study was to analyse the relationship between the DE and ME of typical diets for Korean Hanwoo beef cattle, taking into consideration the gender (male and female) and BW (above and below 350 kg) of the animals.

## 2. Materials and Methods

The data used for the present work were from published research papers and reports. Therefore, the requirement for animal care and use committee approval was waived, because no animals were used in this study.

### 2.1. Database Source and Description

Statistical evaluation of the relationship between DE and ME was performed using published data. The data included individual treatments from three different studies published in Korean Journals and national reports from 1983 to 2013. Data were assembled in a dataset of 141 individual animal measurements from 35 respiration calorimetry experiments with a wide range of diets and energy intake levels. In addition, data were generated from experimental studies carried out in the National Institute of Animal Science (NIAS), Republic of Korea (average data are presented in Table 1). The full database of the individual observations used in the current study is described in the Supplementary Information (Table S1). These papers and reports were chosen, because they reported experiments involving growing steers (*n* = 36) [10,11,12], fattening steers (*n* = 54) [13], growing heifers (*n* = 20) [12] and breeding cows (*n* = 31) [14] in which direct measurements of faecal, urinary and methane losses were made with an indirect respiratory calorimetry chamber [10,14] and head hood chamber system [11,12,13] across a wide range of diet qualities. Specifically, the selected papers represented a wide range of dietary DE (1.70 to 3.51 Mcal/kg DM), ME (1.44 to 3.21 Mcal/kg DM), crude protein (CP, 5.61% to 17.05%), ether extract (EE, 1.05% to 3.28%), neutral detergent fibre (NDF, 30.36% to 72.0%) and acid detergent fibre (ADF, 16.20% to 44.81%) concentrations (DM basis). The dietary GE, DE and ME concentrations were tabulated for each study, and the methane and urine energy concentrations (% of DE) were recorded. The dietary composition information was tabulated for descriptive purposes and to evaluate whether other dietary components affected the relationship between the DE and ME, including the measurements of dietary CP, which were available for all data points. The dietary NDF and ADF concentrations were available for only 80 of the 141 individual animal measurements. In addition, the dietary EE was available for only 60 of the total data points. To estimate the NDF, ADF and EE for the studies that did not provide these data, tabular values for the feed ingredients reported by the Korean Feeding Standards [3] were used to assume the ingredient composition information presented in this paper. Table 2 provides a summary of the mean, minimum, maximum and standard deviations of the key variables in the literature database.

### 2.2. Statistical Analyses

The mixed model methods as described by Littell et al. [15] were used to evaluate the relationship between the dietary DE and ME concentrations. The dietary ME concentration was the dependent variable and was regressed to the dietary DE concentration to evaluate the simple linear regression and to the DE, CP, EE, NDF and ADF in a multiple regression approach. As the database was compiled from multiple published studies that varied in animals, locations, years and experimental conditions, each study was included as a random intercept effect in the model to account for the variations associated with different intercepts in the cited studies.

Similar to Galyean et al. [8], citation-adjusted data were created for each individual animal measurements from the simple linear and multiple regression mixed models, as described by Galyean and Tedeschi [16]. For comparison, simple and multiple regression analyses were also conducted with the unadjusted data. An initial stepwise regression was conducted for testing the potential independent variables (DE, CP, EE, NDF and ADF) with an entry *p*-value of 0.05 to remain in the model. The correlation coefficients between the chemical components (CP, EE, NDF and ADF) and energy contents (GE, DE and ME) of the dataset treatments were analysed using the PROC CORR procedure.

The coefficient of determination (R^2^) and root mean square error (RMSE) were determined for all the models. The R^2^ is an indicator that defines the best-fit equations, and the RMSE is an indicator of the model accuracy [17]. The accuracy of the prediction equations for ME concentrations was assessed by regressing the observed ME values from individual animal measurements minus the predicted values for the ME concentrations. The alpha level used for determining the statistical significance was 0.05. All statistical analyses were performed using SAS software (version 9.4; SAS Inst. Inc., Cary, NC, USA).

## 3. Results

### 3.1. Digestible and Metabolizable Energy Relationship Using Linear Regression

**Figure 1:**
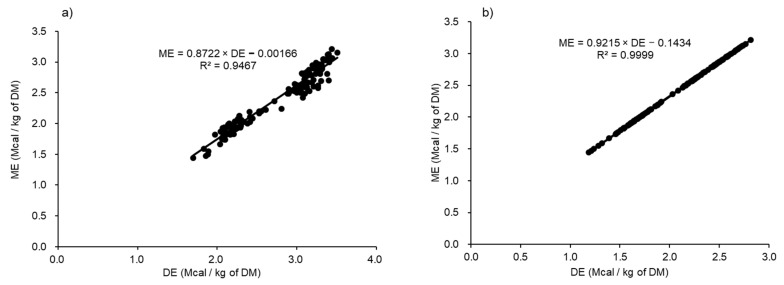
Relationship between the digestible energy (DE) and metabolizable energy (ME) concentrations of the overall data unadjusted (**a**) and adjusted for random differences in intercepts among the citations (**b**) DM; dry matter.

The results of the linear regression analyses of the relationship between ME as a dependent variable and DE as an independent variable using the whole data (unadjusted and adjusted for random coefficients) are graphically represented in Figure 1a. The results showed a strong relationship between the DE and ME across a wide range of dietary conditions, gender and levels of intake (R^2^ = 0.9467 and RMSE = 0.10707, *p* < 0.001 for the intercept and slope; 95% confidence intervals (CI): intercept (–0.0965, 0.0998) and slope (0.8375, 0.9069)), and the linear regression equation was:

ME = 0.8722 × DE − 0.00166(1)

The scatterplot of the adjusted citations for random coefficients presented in Figure 1b showed a similar trend to the unadjusted data, thus improving the R^2^ value (R^2^ = 0.9999 and RMSE = 0.00403), in which the ME and DE were expressed in Mcal/kg of the DM. The 95% CI for the slope and intercept were (–0.2939, 0.0070) and (0.8691, 0.9740), respectively. The adjusted linear regression equation was:

ME = 0.9215 × DE − 0.1434(2)

The ME:DE was about 0.87 at 2.80 Mcal/kg of the DE (approximate mean of the whole dataset).

### 3.2. Digestible and Metabolizable Energy Relationships Between Gender and Body Weight

**Figure 2:**
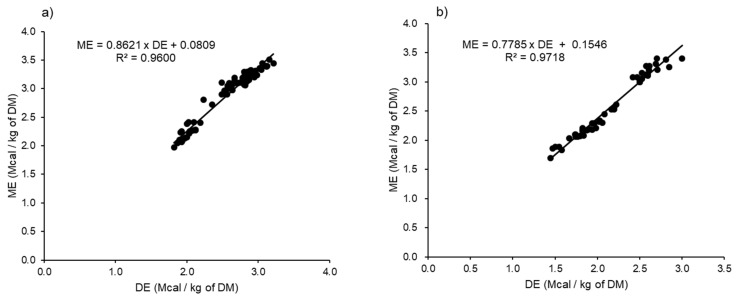
Relationship between the digestible energy (DE) and metabolizable energy (ME) concentrations by gender: male (**a**) and female (**b**) cattle (unadjusted citation for random differences in the intercepts). DM; dry matter.

**Figure 3:**
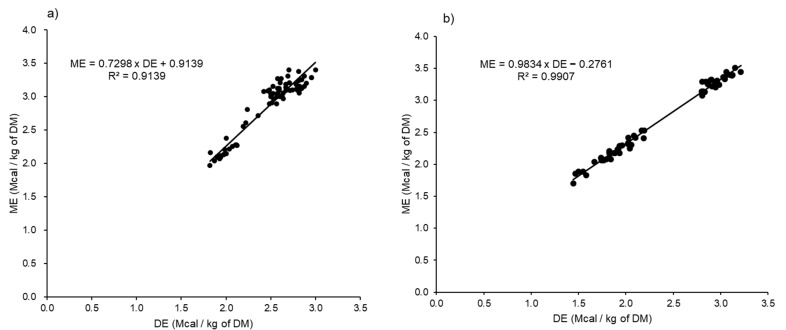
Relationship between the digestible energy (DE) and metabolizable energy (ME) concentrations by body weight: below 350 kg (**a**) and above 350 kg (**b**) (unadjusted citation for random differences in the intercepts). DM; dry matter.

The unadjusted DE and ME relationship between the gender (steer and female cattle) and BW (<350 kg and >350 kg) are plotted in Figure 2 and Figure 3, respectively. By gender, the results showed a strong relationship between the DE and ME, with R^2^ values of 0.9600 and 0.9718 for male (*n* = 90) and female (*n* = 51) beef cattle, respectively. The adjusted citation showed increases in the precision of the prediction equations for both male and female cattle, with R^2^ of 0.9929 and 0.9999, respectively. The steers showed a higher ME:DE (0.89) compared to female cattle (0.83). By BW, the unadjusted data showed a strong positive correlation between the DE and ME, where the R^2^ of the generated equations were 0.9907 and 0.9139 for animals with BW < 350 kg and >350 kg, respectively. The adjusted data improved the R^2^ of the ME prediction equations to 0.9998 and 0.9999, respectively. In addition, the results showed a difference in the ME:DE between animals with BW below and above 350 kg (0.86 and 0.88, respectively). The unadjusted and adjusted linear regression equations expressing the DE and ME relations for animals by gender and BW are shown in Table 3.

### 3.3. Digestible and Metabolizable Energy Relationship Considering Diet Nutrients

**Figure 4:**
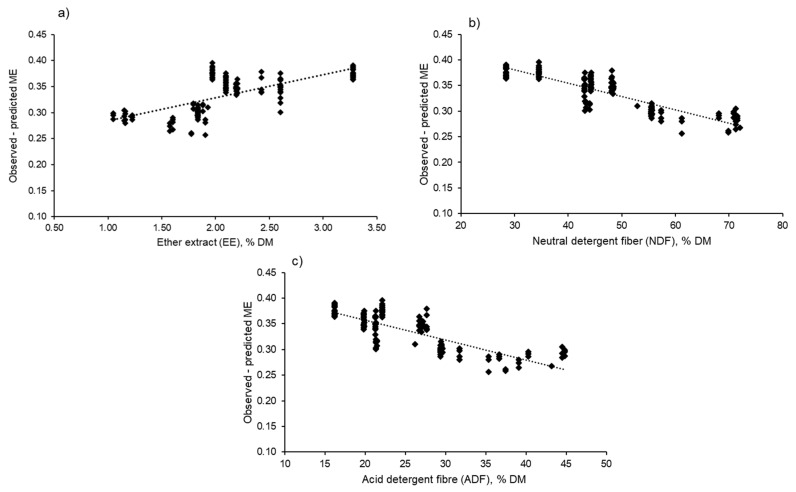
Residual analyses (*n* = 141) of the predicted ME using simple linear regression (Equation (2)) with the EE (**a**), NDF (**b**) and ADF (**c**). DM; dry matter.

The Pearson correlation coefficients (r) of the various chemical compositions (CP, EE, NDF and ADF) to the GE, DE and ME contents of the dataset treatments are presented in Table 4. The ME showed strong negative correlations (*p* < 0.001) with the NDF and ADF dietary contents. In addition, the ME showed strong positive correlations with the DE, EE and CP dietary contents. Specific dietary nutrients that modify the ruminal fermentation may affect the relationship between the DE and ME. The results of the residual analyses (*n* = 141) of the predicted ME using a simple linear regression with EE, NDF and ADF are presented in Figure 4. The evaluation of the prediction errors of the ME (observed minus predicted ME) using Equation (2) showed strong correlations (*p* < 0.001) of the EE (r = 0.67), NDF (r = –0.87) and ADF (r = –0.79) with the ME prediction errors, suggesting that the ME prediction will probably be biased by these dietary factors, and their inclusion could improve the precision of the predictive equations.

Multiple regression analyses were performed to determine the extents of the effects of the dietary components compared to the DE as a single independent variable. The results of the stepwise regression analyses showed that only the CP, NDF and ADF concentrations introduced as independent variables into the model along with the DE were significant (*p* < 0.001) for predicting the ME. However, the EE concentration in the diet as an independent factor was not significant (*p* > 0.05) (Table 5). Therefore, we finally removed this variable (EE, % DM) from the predictive equation, and the final equation was: ME = 1.149 × DE − 0.045 × CP + 0.011 × NDF − 0.027 × ADF + 0.683, Equation (9).

The DE and ME were expressed in Mcal/kg of the DM, and the CP, NDF and ADF were expressed as percentages of the DM (R^2^ = 0.9621); the intercept and slope coefficients in the model were significant at *p* < 0.001 (95% CI: intercept (0.1269; 1.0447), DE (1.0748; 1.2242), CP (–0.0571; –0.0314), NDF (0.0064; 0.0169) and ADF (–0.0354; –0.0169)).

### 3.4. Evaluation and Validation of the DE and ME Relationship Equation

**Figure 5:**
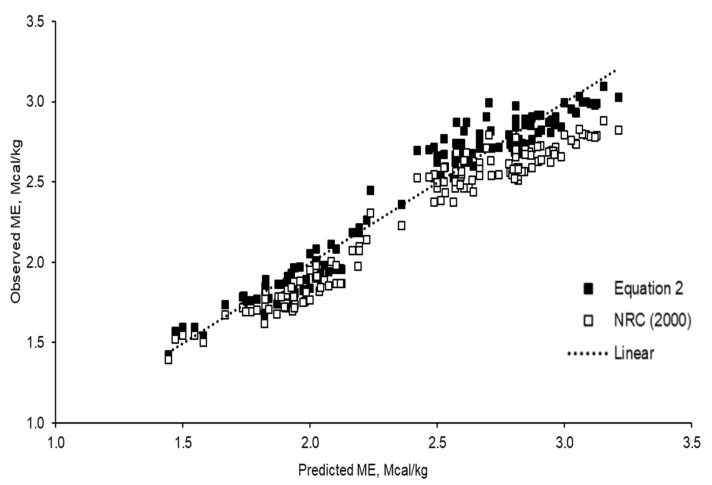
Relationship between the observed ME and predicted ME from the DE using DE × 0.82 (NRC, 2000) and using Equation (2) (ME = 0.9215 × DE − 0.1434) generated in this study.

Figure 5 summarises the statistics of the prediction errors of the ME (observed ME minus the predicted ME) using the whole dataset from Equation (2) generated in the present study and the previously published model proposed by NRC (2000). The results in Figure 5 suggest that both equations were precise, but the precision of Equation (2) was higher (R^2^ = 0.9995) than the NRC equation (ME = 0.82 × DE; R^2^ = 0.9461). In addition, Equation (2) was more accurate and had a lower RSME (0.0059) than the NRC equation (RSME = 0.0079).

## 4. Discussion

This study highlighted the importance of updating the NRC equation used for converting the DE to ME in the Hanwoo beef industry. The data analysed represented a broad range in terms of animal age (growing animals, early and later fattening steers and breeding cows); BW and diet type (forage and concentrate feeds) of Hanwoo beef cattle. This study showed that the ME:DE is higher than the fixed NRC conversion factor (0.82). This equation seems to not be adequate for Hanwoo beef cattle, which led to overfeeding animals to meet their energy requirements. Our DE and ME values were similar to those reported by Galyean and Tedeschi [16] for several beef cattle breeds (Friesian, Hereford, Brahman, Thai, Angus, Hereford × Angus, Jersey, MARC II composite, Holstein, Charolais-cross and Continental × British), ranging from 1.76 to 3.83 Mcal/kg of DM and 1.4 to 3.5 Mcal/kg of DM, respectively. Hales [7] reported higher DE (range from 1.8 to 4.6 Mcal/kg of DM) and ME values (maximised to be between 3.43 and 3.65 Mcal ME/kg of the DM intake) of different diet feedlots, particularly high-concentrate diets, for beef cattle production in the USA.

We found a strong relationship between the DE and ME for gender and BW. This relationship was similar to that of Galyean et al. [8], who reviewed the literature of DE and ME from 87 dietary treatments and found a strong relationship between the DE and ME. The generated equation from the Galyean et al. [8] study was 0.96 × DE − 0.3 (expressed in Mcal/kg), where the ME was about 87% of the DE% (at 2.80 Mcal/kg of DE, an approximate mean of the whole dataset) and not the fixed conversion ratio of 82% recommended by the NRC [4,6]. Our ME:DE conversion factor results were similar to those reported by Galyean et al. [8] calculated from the combination of several beef cattle breeds. There has been substantial discussion regarding the limitations of the constant value of 0.82 recommended by the NRC to convert DE to ME in beef cattle, and it has been recommended that it should be updated, taking into consideration the genotype, diet composition, gender and age of the animals [7,8,9,17,18]. Furthermore, Kim et al. [10] estimated the ME:DE of Hanwoo steers weighing 376.6 and fed rice straw (44%) and concentrate (56%) at three different energy levels (0.8, 1.2 and 1.6 times the maintenance), and the ME:DE ranged between 83% and 90%.

The Agriculture and Food Research Council (AFRC) [19] suggests that converting DE to ME could be affected by several factors, such as the body weight and weight change (kg per day); growth maturity (early, medium and late stages) and gender (bulls, castrates and heifers).

We observed differences in energy utilisation according to gender. This difference may reflect differences in the energy costs of the maintenance and production of beef cattle of different physiological status and productivity. Our ME:DE were 0.89 in males and 0.83 in females. The steers were more efficient in converting DE to ME compared to female cattle because of the specific feeding program for steers where the animals received high metabolic diets, in particular, to produce high fat depositions in the intramuscular tissues. Steers were fed diets with a forage-to-concentrate ratio ranging from 40:60 in the growing phase (7–13 months of age) to 30:70 in the early fattening phase (14–21 months of age) and 10:90 in the late fattening phase (22–30 months of age) [20,21]. Furthermore, high-quality grass hays were supplied from the growing to the early fattening phases, and rice straw was given in the late fattening phase. Furthermore, rice straw is a major roughage source, accounting for around 60% of the daily DM intake in the life cycle of breeding cows [3]. Differences in the ME:DE are mostly due to differences of the BW gains, resulting in differences in the feed efficiency and in the efficiency of ME utilisation [22]. Valente et al. [23] found a similar trend in the estimation of the energy requirements of Nellore beef cattle in a tropical pasture. 

Moreover, the BW is another key driver determining the relationship between the DE and ME in the feeding management of Hanwoo cattle. A BW of 350 kg at around 13 months of age is the typical switching point from the growing to the fattening phase for Hanwoo beef cattle in Korea [21], and thereafter, the ratio of the daily retained energy content-to-daily gain markedly increases until 30 months of age [24]. In addition, a BW of 350 kg is the beginning stage for increasing the rates of accumulation of the lipids in Hanwoo beef cattle not only intramuscularly but, also, in the subcutaneous tissue by gradually supplying high-concentrate diets. Breeding heifers are considered to have reached sexual maturity at 14 to 15 months of age (range from 300- to 350-kg BW). In our study, animals with BW above 350 kg had a higher ME:DE conversion factor (88%) than those below 350 kg (86%). However, there has been some controversy about its influence on the ME:DE, which is generally greater in growing than mature ruminants, and this has been correlated with less methane and urinary loss in growing ruminants [7,9]. It is also generally recognised that methane emissions are lower for mature cattle fed high-concentrate-based diets compared to growing cattle fed high-forage-based diets [25,26,27]. The efficiency of converting metabolizable protein to net protein was proposed by Fox et al. [1] as 75% for BW ranging from 0 to 181 kg, 50% for BW between 181 and 360 kg and 40% for BW more than 363 kg. Similarly, Kim et al. [24] reported that the protein content with a daily BW gain for Hanwoo steers decreased from 18% to 7% as the growing steers became older. While the efficiency of the deposition of energy as fat is higher than as protein [19,28], Hanwoo is one beef cattle breed that is able to produce highly marbled meat, and this ability starts functioning from 12 months of age. Lipogenesis is accelerated with advancing maturity [29]. Kwon et al. [30] reported that Hanwoo beef cattle has a high marbling score compared to beef cattle produced USA and Australian meat. Therefore, Hanwoo beef cattle has gained more available energy for fat deposition than did cattle of other breeds reported by Galyean et al. [8], which explains the high efficiency of converting the ME to DE in Hanwoo cattle. Nonetheless, methane and urinary energy seem not to be appropriate explanations for mature Hanwoo in the fattening phase being fed high-concentrate-based diets with a percentage of inclusion that ranged from 70% to 90%. There are sufficient data in the literature to directly compare the ME:DE ratio in response to increased inclusion of the concentrate in the diet [7,19,31]. Similarly, Fuller et al. [31] reported that the efficiency of the DE to ME conversion of Angus steers increased quadratically (*p* < 0.01) as the forage-to-concentrate ratio decreased, 0.86 to 0.92.

Galyean et al. [8] reported that other dietary factors could change the ruminal fermentation and, consequently, affect the linear relationship between the DE and ME. However, although our results indicate that the ME is highly correlated with the fibre content (NDF and ADF) and EE (*p* < 0.001), initial analyses using stepwise regression indicated that only the CP, NDF and ADF concentrations in the diet were significantly independent variables, along with the DE (*p* < 0.001), for predicting the dietary ME (Equation (9)). This study did not consider the data points of the starch content, as some trials did not report the starch content in the diet. Moreover, the results showed that the inclusion of dietary factors did not improve the accuracy of the prediction of the ME (R^2^ = 0.9621) compared to the estimation of the ME using a single-variable (DE) regression approach (R^2^ = 0.9999).

A comparative slaughter studydetermined the net energy requirements for the growth of Hanwoo steers from 6 to 30 months of age [24]. Eight steers were randomly selected from 104 steers every 2 months, and subsamples of the whole empty body components were analysed for body composition determination. The Korean Feeding Standards [3] used an efficiency of 47% to convert the net energy into metabolizable energy. The evaluation of the observed vs. predicted ME concentrations using the fixed ME:DE of 0.82 from the NRC [6] and from Equation (2) generated in this study were accurate (R^2^ > 0.9000). However, Equation (2) gave a higher prediction precision in terms of the R^2^ (0.9995) compared to the NRC (2000) equation (R^2^ = 0.9461). Therefore, Equation (2) has higher predictive power than the previously published ME:DE [6] currently adopted in the energy system for Korean Hanwoo beef cattle [3]. Similar to Galyean et al. [8], the fixed ME:DE of 0.82 could be used for low-quality diets, while Equation (2) may be suitable for diets with higher ME concentrations. Therefore, it is recommended to use upgraded ME, DE and total digestible nutrients, as well as dry matter requirements, for Hanwoo beef cattle.

## 5. Conclusions

This study highlighted the importance of updating the relationship between the DE and ME recommended by the NRC using Hanwoo beef cattle data. Our results strongly suggest that the use of a constant value of 0.82 to convert the dietary concentration of DE into ME for beef cattle diets should be replaced with a new model that considers the national specificity of animal performances and the nature of the diet energy contents. Therefore, it is recommended to adopt the newly generated equation (ME = 0.9215 × DE − 0.1434) for Hanwoo beef cattle instead of the NRC ME:DE constant conversion factor of 0.82. Our results will be useful for future studies that aim to update the energy requirements for the maintenance and growth of beef cattle in Korea. Future research is warranted to re-evaluate the ME:DE conversion factor of cattle in other regions and continents according to the specificity of the breed and feed quality in order to understand the underlying mechanisms that affect the conversion of DE to ME.

## Figures and Tables

**Table 1:** Database of the average observations used for evaluation of the relationship between the DE and ME.

						Percentage of DM				Mcal/kg of DM				Percentage of DE		
Source	Diet	Animal	Nb Ob	BW, kg	DMI, kg/Day	CP	EE	NDF	ADF	GE	DE	ME	ME:DE	CH_4_	Urine	Method ^1^
[14]	Mixed orchard hay I	First calving cows ^2^	4	392	4.63	9.47	1.16	57.35	31.72	4.62	2.22	1.89	0.8487	10.5	4.2	HHCS
[14]	Mixed orchard hay II	First calving cows	2	377	5.05	6.57	1.77	69.90	37.42	3.98	1.87	1.49	0.7936	17.3	4.2	HHCS
[14]	Mixed orchard hay III	First calving cows	1	366	5.30	6.82	1.60	72.00	43.19	4.17	1.83	1.58	0.8660	10.4	2.1	HHCS
[14]	Orchard hay III + Rice straw I	First calving cows	3	347	4.40	6.10	1.60	71.53	36.65	4.13	2.12	1.82	0.8603	10.7	2.9	HHCS
[14]	Orchard hay III + Rice straw I	First calving cows	3	352	5.33	5.61	1.58	71.29	39.09	4.05	2.01	1.65	0.8223	14.9	3.1	HHCS
[14]	Orchard hay IV + Rice straw II	First calving cows	3	330	5.30	7.13	1.15	71.21	44.46	4.37	2.19	1.92	0.8759	9.6	3.1	HHCS
[14]	Orchard hay IV + Rice straw II	First calving cows	3	319	6.20	6.68	1.05	70.81	44.81	4.32	2.16	1.92	0.8901	8.8	2.0	HHCS
[14]	Orchard hay III + Rice straw I + Wheat bran I	First calving cattle	3	351	6.30	6.67	1.90	61.15	35.37	3.86	1.99	1.67	0.8413	12.6	3.4	HHCS
[14]	Orchard hay IV + Rice straw II + Wheat bran II	First calving cows	3	317	6.20	7.67	1.23	68.05	40.25	4.34	2.21	1.88	0.8517	11.8	3.1	HHCS
[14]	Orchard hay IV + Concentrate II	First calving cows	3	291	3.60	12.06	1.80	43.30	21.44	4.47	2.54	2.16	0.8540	10.9	4.0	HHCS
[14]	Orchard hay III + Concentrate I	First calving cows	3	341	3.60	11.19	1.88	43.98	21.35	4.58	2.46	2.13	0.8647	10.9	2.6	HHCS
[11]	Timothy hay + Barley	Growing steer ^3^	6	170	2.10	14.14	2.10	44.27	19.88	4.10	3.13	2.69	0.8601	11.7	2.8	HHCS
[11]	Timothy hay + Corn	Growing steer	6	173	2.10	14.57	2.60	43.08	21.17	4.14	2.97	2.55	0.8562	11.7	2.7	HHCS
[11]	Timothy hay + Barley	Growing steer	6	178	3.20	14.15	2.10	44.23	19.85	4.28	3.09	2.70	0.8733	9.8	2.9	HHCS
[11]	Timothy hay + Corn	Growing steer	6	178	3.30	14.58	2.60	43.04	21.14	4.24	3.04	2.70	0.8868	8.6	3.0	HHCS
[11]	Timothy hay + Barley	Growing steer	6	187	3.90	14.15	2.10	44.23	19.85	4.31	3.15	2.74	0.8708	9.1	3.8	HHCS
[11]	Timothy hay + Corn	Growing steer	6	183	3.90	14.58	2.60	43.04	21.14	4.21	2.96	2.61	0.8824	8.7	3.4	HHCS
[13]	Barley + rice straw	Late fattening steer	4	602	5.73	17.05	1.97	48.31	22.14	4.25	3.30	2.99	0.9051	6.2	3.4	HHCS
[13]	Corn + rice straw	Late fattening steer ^4^	5	608	5.62	16.70	3.28	30.36	16.20	4.31	3.30	2.97	0.9015	6.0	3.9	HHCS
[13]	Barley + rice straw	Late fattening steer	5	621	6.88	17.05	1.97	48.31	22.14	4.23	3.32	3.01	0.9063	5.5	3.9	HHCS
[13]	Corn + rice straw	Late fattening steer	5	605	6.62	16.70	3.28	30.36	16.20	4.32	3.32	2.97	0.8927	6.1	4.5	HHCS
[13]	Barley + rice straw	Late fattening steer	5	615	7.80	17.05	1.97	48.31	22.14	4.24	3.24	2.94	0.9082	5.9	3.3	HHCS
[13]	Corn + rice straw	Late fattening steer	6	616	7.65	16.70	3.28	30.36	16.20	4.29	3.32	2.94	0.8870	7.3	4.1	HHCS
[12]	Rice straw + Concentrate III	Growing heifers ^5^	2	154	2.40	10.46	2.43	48.19	27.66	4.11	3.02	2.50	0.8276	9.8	7.6	HHCS
[12]	Rice straw + Concentrate III	Growing heifers	2	148	3.45	10.53	2.43	48.19	27.66	4.24	2.95	2.53	0.8572	10.2	4.0	HHCS
[12]	Rice straw + Concentrate III	Growing heifers	2	170	3.80	10.53	2.43	48.19	27.66	4.32	3.34	2.93	0.8775	7.7	4.8	HHCS
[12])	Rice straw + Concentrate IV	Heifers ^6^	3	213	2.60	9.86	2.19	48.47	31.18	4.35	3.28	2.63	0.8007	11.5	8.2	HHCS
[12]	Rice straw + Concentrate IV	Heifers	5	207	3.80	9.89	2.19	48.30	31.00	4.26	3.14	2.56	0.8139	10.3	8.5	HHCS
[12]	Rice straw + Concentrate IV	Heifers	6	207	4.70	9.95	2.20	48.00	30.66	4.30	3.21	2.63	0.8188	9.4	8.6	HHCS
[12]	Rice straw + Concentrate V	Steer ^7^	6	224	4.60	12.65	1.84	55.49	29.37	3.75	2.10	1.94	0.9246	7.1	0.5	HHCS
[12]	Rice straw + Concentrate V	Steer	6	207	3.40	12.89	1.85	55.06	28.85	3.76	2.17	2.00	0.9250	7.0	0.6	HHCS
[12]	Rice straw + Concentrate V	Steer	6	187	2.27	12.62	1.84	55.55	29.44	3.72	2.16	1.98	0.9178	8.0	0.2	HHCS
[10]	Rice straw + Concentrate VI	Steer	2	371	7.30	12.62	1.84	55.54	29.42	3.94	2.33	2.12	0.9088	7.7	1.7	HHCS
[10]	Rice straw + Concentrate VI	Steer	2	391	5.70	12.60	1.83	55.59	29.48	3.94	2.32	2.01	0.8637	12.1	0.8	HHCS
[10]	Rice straw + Concentrate VI	Steer	2	371	4.05	12.55	1.83	55.67	29.59	3.99	2.33	1.98	0.8470	13.2	2.6	HHCS

^1^ HHCS, head hood chamber system. ^2^ First calving cows refers to Hanwoo cows getting their first calving at 24 months of age. ^3^ Growing steers stage refers to animals between 7 and 13 months of age. ^4^ Late fattening steers stage refers to animals between 22 to 30 months of age. ^5^ The growing heifers stage refers to animals between 7 and 13 months of age. Hanwoo heifers refers to female animals that attain sexual maturity at 14 months of age. ^7^ Hanwoo steers refers to animals in the early fattening stage between 14 and 21 months of age. ADF, acid detergent fibre; CP, crude protein; DE, digestible energy; EE, ether extract; GE, gross energy; ME, metabolizable energy; CH_4_, methane; NDF, neutral detergent.

**Table 2:** Variability of the chemical components of the diets (DM basis), DM intake and animal BW in the database.

Database	Mean	Minimum	Maximum	SD
Literature database				
No. of observations	141	-	-	-
BW, kg	321	146	650	168.2
DMI, kg/d	4.55	2.00	7.90	1.7
CP, %	12.60	5.61	17.05	3.4
EE, %	2.13	1.05	3.28	0.6
NDF, %	49.45	30.36	72.00	11.0
ADF, %	26.27	16.20	44.81	7.6
GE, Mcal/kg	4.18	3.43	4.65	0.2
DE, Mcal/kg	2.78	1.70	3.51	0.5
ME, Mcal/kg	2.43	1.44	3.21	0.5
ME:DE	0.87	0.79	0.93	0.0
CH_4_, % DE *	9.2	4.2	18.30	2.8
Urine, % DE *	3.5	0.1	11.3	2.3

ADF, acid detergent fibre; CP, crude protein; DE, digestible energy; EE, ether extract; GE, gross energy; ME, metabolizable energy; NDF, neutral detergent fibre; SD, standard deviation. * The ratio of CH_4_ and urine energy losses to the DE energy intake.

**Table 3:** Adjusted and unadjusted equations of the relationships between the digestible and metabolizable energy of beef cattle according to gender and body weight.

Parameter	Categories	*n*	ME:DE	Unadjusted Citation			Adjusted Citation		
				Equation	R^2^	RSME	Equation	R^2^	RSME
Gender	Steer	90	0.89	ME = 0.8621 × DE + 0.0809 Equation (1a)	0.9600	0.08303	ME = 0.9696 × DE − 0.2140 Equation (2a)	0.9929	0.00384
	Female	51	0.83	ME = 0.7785 × DE + 0.1546 Equation (3)	0.9718	0.07083	ME = 0.9397 × DE − 0.2951 Equation (4)	0.9999	0.00349
BW	<350 kg	63	0.88	ME = 0.9834 × DE − 0.2761 Equation (5)	0.9907	0.05539	ME = 0.9834 × DE − 0.2761 Equation (6)	0.9998	0.00396
	>350 kg	78	0.86	ME = 0.7298 × DE + 0.9139 Equation (7)	0.9139	0.10052	ME = 0.9696 × DE − 0.2140 Equation (8)	0.9999	0.00403

BW, body weight; DE, digestible energy; ME, metabolizable energy.

**Table 4:** Correlation coefficients among the chemical compositions and energy in the diets.

Item	GE	DE	ME	CP	EE	NDF	ADF
GE	1.00						
DE	0.47	1.00					
ME	0.40	0.97 ***	1.00				
CP	−0.07	0.52	0.59	1.00			
EE	0.11	0.67 ***	0.67 ***	0.57	1.00		
NDF	−0.30	−0.83 ***	−0.87 ***	−0.72 ***	−0.80 ***	1.00	
ADF	−0.22	−0.78 ***	−0.79 ***	−0.84 ***	−0.79 ***	0.94 ***	1.00

ADF, acid detergent fibre; CP, crude protein; DE, digestible energy; EE, ether extract; GE, gross energy; ME, metabolizable energy; NDF, neutral detergent fibre. *** *p* < 0.001.

**Table 5:** Metabolizable energy prediction from the digestible energy and feed nutrients using multiple regression analyses.

Variable	Parameter Estimate	95% CI	*p*-Value
Intercept	0.683	(0.1269, 1.0447)	0.0007
DE	1.149	(1.0748, 1.2242)	<0.0001
CP	−0.045	(−0.0571, −0.0314)	<0.0001
EE	0.020	(−0.0309, 0.0719)	0.4323
NDF	0.011	(0.0064, 0.0169)	<0.0001
ADF	−0.027	(−0.0354, −0.0169)	<0.0001

ADF, acid detergent fibre; CP, crude protein; CI, confidence interval; DE, digestible energy; EE, ether extract; ME, metabolizable energy; NDF, neutral detergent fibre.

## Data Availability

The data presented in this study are available on request from the corresponding author.

## Supplementary Materials

The following are available online at https://www.mdpi.com/article/10.3390/ani11061696/s1; Table S1. Database of individual ME.
