# Supplementary material for: Developing Equations for Converting Digestible Energy to Metabolizable Energy for Korean Hanwoo Beef Cattle
Source: Animals (Basel). 2021 Jun 7;11(6):1696. doi: 10.3390/ani11061696 (PMC8228101; doi:10.3390/ani11061696)
Supplement: Supplementary file 1 [file animals-11-01696-s001.zip › animals-1190744-supplementary.pdf]

Supplementary Information (Table S1)

Table S1. Database of individual observations used for evaluation of the relationship between DE and ME.

Source	Diet	Animal	Nb Ob	BW, kg	DMI, kg/d	Percentage of DM				Mcal/kg of DM			ME:DE	Percentage of DE		Method[1]
						CP	EE	NDF	ADF	GE	DE	ME		CH_4	Urine	
[1]	Mixed orchard hay I	First calving cows	1	407.0	5.1	9.47	1.16	57.35	31.72	4.61	2.29	1.96	0.8547	10.1	4.3	HHCS
[1]	Mixed orchard hay I	First calving cows	1	430.0	4.4	9.47	1.16	57.35	31.72	4.61	2.32	2.02	0.8725	8.9	3.9	HHCS
[1]	Mixed orchard hay I	First calving cows	1	394.0	5.0	9.47	1.16	57.35	31.72	4.60	2.10	1.74	0.8286	12.6	3.8	HHCS
[1]	Mixed orchard hay I	First calving cows	1	335.0	4.0	9.47	1.16	57.35	31.72	4.65	2.18	1.83	0.8391	10.4	4.6	HHCS
[1]	Mixed orchard hay II	First calving cows	1	353.0	5.2	6.57	1.77	69.90	37.42	4.00	1.88	1.50	0.7959	16.3	5.1	HHCS
[1]	Mixed orchard hay II	First calving cows	1	400.0	4.9	6.57	1.77	69.90	37.42	3.96	1.86	1.47	0.7912	18.3	3.3	HHCS
[1]	Mixed orchard hay III	First calving cows	1	366	5.30	6.82	1.60	72.00	43.19	4.17	1.83	1.58	0.8660	10.4	2.1	HHCS
[1]	Orchard hay III + Rice straw I	First calving cows	1	347.0	4.7	6.10	1.60	71.53	36.65	4.13	2.06	1.77	0.8557	11.2	3.1	HHCS
[1]	Orchard hay III + Rice straw I	First calving cows	1	345.0	4.0	6.10	1.60	71.53	36.65	4.13	2.18	1.88	0.8621	10.1	3.4	HHCS
[1]	Orchard hay III + Rice straw I	First calving cows	1	350.0	4.5	6.10	1.60	71.53	36.65	4.13	2.11	1.82	0.8632	10.9	2.1	HHCS
[1]	Orchard hay III + Rice straw I	First calving cows	1	353.0	5.4	5.61	1.58	71.29	39.09	4.07	2.04	1.67	0.8182	16.1	2.7	HHCS
[1]	Orchard hay III + Rice straw I	First calving cows	1	359.0	5.3	5.61	1.58	71.29	39.09	4.06	2.09	1.74	0.8288	14.5	2.7	HHCS
[1]	Orchard hay III + Rice straw I	First calving cows	1	344.0	5.3	5.61	1.58	71.29	39.09	4.02	1.89	1.55	0.8200	14.1	4.0	HHCS
[1]	Orchard hay IV + Rice straw II	First calving cows	1	353.0	5.3	7.13	1.15	71.21	44.46	4.36	2.08	1.79	0.8636	11.2	2.7	HHCS
[1]	Orchard hay IV + Rice straw II	First calving cows	1	335.0	5.3	7.13	1.15	71.21	44.46	4.36	2.19	1.91	0.8707	9.7	3.4	HHCS
[1]	Orchard hay IV + Rice straw II	First calving cows	1	302.0	5.3	7.13	1.15	71.21	44.46	4.40	2.30	2.06	0.8934	7.9	3.3	HHCS

[1]	Orchard hay IV + Rice straw II	First calving cows	1	341.0	6.2	6.68	1.05	70.81	44.81	4.32	2.18	1.94	0.8889	9.5	1.5	HHCS
[1]	Orchard hay IV + Rice straw II	First calving cows	1	326.0	6.2	6.68	1.05	70.81	44.81	4.32	2.08	1.84	0.8837	9.1	2.3	HHCS
[1]	Orchard hay IV + Rice straw II	First calving cows	1	290.0	6.2	6.68	1.05	70.81	44.81	4.32	2.21	1.98	0.8978	7.9	2.2	HHCS
[1]	Orchard hay III + Rice straw I + Wheat bran I	First calving cows	1	350.0	6.3	6.67	1.90	61.15	35.37	4.08	2.06	1.75	0.8462	11.0	4.6	HHCS
[1]	Orchard hay III + Rice straw I + Wheat bran I	First calving cows	1	352.0	6.3	6.67	1.90	61.15	35.37	4.08	2.21	1.83	0.8273	14.6	2.9	HHCS
[1]	Orchard hay III + Rice straw I + Wheat bran I	First calving cows	1	350.0	6.3	6.67	1.90	61.15	35.37	3.43	1.70	1.44	0.8505	12.3	2.8	HHCS
[1]	Orchard hay IV + Rice straw II + Wheat bran II	First calving cows	1	346.0	6.2	7.67	1.23	68.05	40.25	4.34	2.18	1.89	0.8667	11.1	2.2	HHCS
[1]	Orchard hay IV + Rice straw II + Wheat bran II	First calving cows	1	316.0	6.2	7.67	1.23	68.05	40.25	4.34	2.29	1.94	0.8451	12.5	3.5	HHCS
[1]	Orchard hay IV + Rice straw II + Wheat bran II	First calving cows	1	289.0	6.2	7.67	1.23	68.05	40.25	4.34	2.16	1.82	0.8433	11.8	3.7	HHCS
[1]	Orchard hay IV + Concentrate II	First calving cows	1	314.0	3.6	12.06	1.80	43.30	21.44	4.47	2.44	2.08	0.8523	10.8	4.5	HHCS
[1]	Orchard hay IV + Concentrate II	First calving cows	1	294.0	3.6	12.06	1.80	43.30	21.44	4.47	2.56	2.19	0.8587	9.7	5.4	HHCS
[1]	Orchard hay IV + Concentrate II	First calving cows	1	266.0	3.6	12.06	1.80	43.30	21.44	4.47	2.61	2.22	0.8511	12.2	2.1	HHCS
[1]	Orchard hay III + Concentrate I	First calving cows	1	320.0	3.6	11.19	1.88	43.98	21.35	4.58	2.53	2.17	0.8571	11.8	2.2	HHCS
[1]	Orchard hay III + Concentrate I	First calving cows	1	318.0	3.6	11.19	1.88	43.98	21.35	4.58	2.53	2.19	0.8681	9.9	3.3	HHCS
[1]	Orchard hay III +	First calving cows	1	384.0	3.6	11.19	1.88	43.98	21.35	4.58	2.33	2.03	0.8690	11.1	2.4	HHCS

	Concentrate I															
[2]	Timothy hay + Barley	Growing steer	1	163.0	2.1	14.14	2.10	44.27	19.88	4.10	3.19	2.67	0.8358	14.0	3.0	HHCS
[2]	Timothy hay + Barley	Growing steer	1	165.0	2.1	14.14	2.10	44.27	19.88	4.10	3.10	2.71	0.8769	8.6	4.6	HHCS
[2]	Timothy hay + Barley	Growing steer	1	178.0	2.1	14.14	2.10	44.27	19.88	4.10	3.00	2.57	0.8571	10.9	3.2	HHCS
[2]	Timothy hay + Barley	Growing steer	1	176.0	2.1	14.14	2.10	44.27	19.88	4.10	3.14	2.67	0.8485	12.8	3.0	HHCS
[2]	Timothy hay + Barley	Growing steer	1	175.0	2.1	14.14	2.10	44.27	19.88	4.10	3.29	2.95	0.8986	9.4	1.4	HHCS
[2]	Timothy hay + Barley	Growing steer	1	163.0	2.1	14.14	2.10	44.27	19.88	4.10	3.05	2.57	0.8438	14.6	1.6	HHCS
[2]	Timothy hay + Corn	Growing steer	1	167.0	2.1	14.57	2.60	43.08	21.31	4.14	3.10	2.67	0.8615	11.7	3.1	HHCS
[2]	Timothy hay + Corn	Growing steer	1	202.0	2.1	14.57	2.60	43.08	21.31	4.14	3.24	2.81	0.8676	11.1	2.9	HHCS
[2]	Timothy hay + Corn	Growing steer	1	173.0	2.1	14.57	2.60	43.08	21.31	4.14	2.38	2.00	0.8400	11.7	4.0	HHCS
[2]	Timothy hay + Corn	Growing steer	1	171.0	2.1	14.57	2.60	43.08	21.31	4.14	3.29	2.95	0.8986	8.6	1.4	HHCS
[2]	Timothy hay + Corn	Growing steer	1	165.0	2.1	14.57	2.60	43.08	21.31	4.14	3.00	2.62	0.8730	9.8	1.6	HHCS
[2]	Timothy hay + Corn	Growing steer	1	162.0	2.1	14.57	2.60	43.08	21.31	4.14	2.81	2.24	0.7966	17.4	3.4	HHCS
[2]	Timothy hay + Barley	Growing steer	1	181.0	3.2	14.15	2.10	44.23	19.85	4.28	3.16	2.81	0.8911	9.7	2.0	HHCS
[2]	Timothy hay + Barley	Growing steer	1	183.0	3.2	14.15	2.10	44.23	19.85	4.28	3.09	2.59	0.8384	12.9	3.0	HHCS
[2]	Timothy hay + Barley	Growing steer	1	165.0	3.2	14.15	2.10	44.23	19.85	4.28	2.97	2.53	0.8526	10.2	4.2	HHCS
[2]	Timothy hay + Barley	Growing steer	1	166.0	3.2	14.15	2.10	44.23	19.85	4.28	3.13	2.84	0.9100	6.8	2.0	HHCS
[2]	Timothy hay + Barley	Growing steer	1	190.0	3.2	14.15	2.10	44.23	19.85	4.28	3.19	2.78	0.8725	10.8	2.0	HHCS
[2]	Timothy hay + Barley	Growing steer	1	183.0	3.2	14.15	2.10	44.23	19.85	4.28	3.00	2.63	0.8750	8.2	4.2	HHCS
[2]	Timothy hay + Corn	Growing steer	1	174.0	3.3	14.58	2.60	43.04	21.27	4.24	3.12	2.79	0.8932	9.2	1.9	HHCS
[2]	Timothy hay + Corn	Growing steer	1	175.0	3.3	14.58	2.60	43.04	21.27	4.24	2.91	2.52	0.8646	9.2	5.2	HHCS
[2]	Timothy hay + Corn	Growing steer	1	174.0	3.3	14.58	2.60	43.04	21.27	4.24	3.00	2.61	0.8687	9.1	5.1	HHCS
[2]	Timothy hay + Corn	Growing steer	1	186.0	3.3	14.58	2.60	43.04	21.27	4.24	3.06	2.82	0.9208	6.6	1.0	HHCS
[2]	Timothy hay + Corn	Growing steer	1	184.0	3.3	14.58	2.60	43.04	21.27	4.24	3.09	2.82	0.9118	6.8	2.0	HHCS
[2]	Timothy hay + Corn	Growing steer	1	174.0	3.3	14.58	2.60	43.04	21.27	4.24	3.06	2.64	0.8614	10.5	3.0	HHCS
[2]	Timothy hay + Barley	Growing steer	1	203.0	3.9	14.15	2.10	44.23	19.85	4.31	3.31	2.87	0.8682	10.5	2.3	HHCS
[2]	Timothy hay + Barley	Growing steer	1	203.0	3.9	14.15	2.10	44.23	19.85	4.31	3.10	2.49	0.8017	11.3	8.3	HHCS
[2]	Timothy hay + Barley	Growing steer	1	181.0	3.9	14.15	2.10	44.23	19.85	4.31	3.21	2.90	0.9040	7.1	2.4	HHCS
[2]	Timothy hay + Barley	Growing steer	1	183.0	3.9	14.15	2.10	44.23	19.85	4.31	3.10	2.74	0.8843	8.0	3.3	HHCS
[2]	Timothy hay + Barley	Growing steer	1	177.0	3.9	14.15	2.10	44.23	19.85	4.31	3.03	2.59	0.8559	11.4	3.4	HHCS
[2]	Timothy hay + Barley	Growing steer	1	174.0	3.9	14.15	2.10	44.23	19.85	4.31	3.15	2.87	0.9106	6.1	3.3	HHCS
[2]	Timothy hay + Corn	Growing steer	1	190.0	3.9	14.58	2.60	43.04	21.27	4.21	2.97	2.64	0.8879	8.1	3.4	HHCS

[2]	Timothy hay + Corn	Growing steer	1	193.0	3.9	14.58	2.60	43.04	21.27	4.21	3.10	2.79	0.9008	7.6	2.5	HHCS
[2]	Timothy hay + Corn	Growing steer	1	176.0	3.9	14.58	2.60	43.04	21.27	4.21	2.72	2.36	0.8679	9.5	3.8	HHCS
[2]	Timothy hay + Corn	Growing steer	1	174.0	3.9	14.58	2.60	43.04	21.27	4.21	3.15	2.82	0.8943	8.2	2.4	HHCS
[2]	Timothy hay + Corn	Growing steer	1	166.0	3.9	14.58	2.60	43.04	21.27	4.21	2.90	2.56	0.8850	9.3	2.7	HHCS
[2]	Timothy hay + Corn	Growing steer	1	197.0	3.9	14.58	2.60	43.04	21.27	4.21	2.90	2.49	0.8584	9.3	5.3	HHCS
[3]	Barley + rice straw	Late fattening steer	1	590.0	5.9	12.42	1.97	34.56	22.14	4.27	3.41	3.08	0.9055	5.3	4.5	HHCS
[3]	Barley + rice straw	Late fattening steer	1	632.0	5.5	12.42	1.97	34.56	22.14	4.22	3.20	2.95	0.9205	5.7	2.3	HHCS
[3]	Barley + rice straw	Late fattening steer	1	580.0	5.7	12.42	1.97	34.56	22.14	4.26	3.28	2.95	0.8984	6.8	3.7	HHCS
[3]	Barley + rice straw	Late fattening steer	1	604.0	5.8	12.42	1.97	34.56	22.14	4.26	3.31	2.97	0.8958	7.0	3.1	HHCS
[3]	Corn + rice straw	Late fattening steer	1	616.0	5.3	13.22	3.28	28.39	16.20	4.34	3.32	2.91	0.8750	7.2	5.1	HHCS
[3]	Corn + rice straw	Late fattening steer	1	574.0	5.8	13.22	3.28	28.39	16.20	4.31	3.40	3.10	0.9137	7.2	1.5	HHCS
[3]	Corn + rice straw	Late fattening steer	1	646.0	5.6	13.22	3.28	28.39	16.20	4.32	3.41	3.07	0.9005	4.9	4.7	HHCS
[3]	Corn + rice straw	Late fattening steer	1	595.0	5.8	13.22	3.28	28.39	16.20	4.26	3.28	2.97	0.9053	5.1	4.7	HHCS
[3]	Corn + rice straw	Late fattening steer	1	611.0	5.6	13.22	3.28	28.39	16.20	4.30	3.07	2.80	0.9128	5.7	3.5	HHCS
[3]	Barley + rice straw	Late fattening steer	1	629.0	6.6	12.42	1.97	34.56	22.14	4.26	3.27	2.86	0.8750	7.1	5.6	HHCS
[3]	Barley + rice straw	Late fattening steer	1	643.0	6.8	12.42	1.97	34.56	22.14	4.24	3.34	3.04	0.9119	5.1	3.5	HHCS
[3]	Barley + rice straw	Late fattening steer	1	609.0	7.0	12.42	1.97	34.56	22.14	4.21	3.44	3.21	0.9336	5.3	1.7	HHCS
[3]	Barley + rice straw	Late fattening steer	1	581.0	7.0	12.42	1.97	34.56	22.14	4.21	3.40	3.13	0.9202	4.4	3.8	HHCS
[3]	Barley + rice straw	Late fattening steer	1	641.0	7.0	12.42	1.97	34.56	22.14	4.21	3.14	2.80	0.8909	5.7	5.0	HHCS
[3]	Corn + rice straw	Late fattening steer	1	572.0	6.6	13.22	3.28	28.39	16.20	4.32	3.27	2.94	0.8981	6.0	4.2	HHCS
[3]	Corn + rice straw	Late fattening steer	1	645.0	6.5	13.22	3.28	28.39	16.20	4.31	3.29	2.85	0.8645	8.5	5.1	HHCS
[3]	Corn + rice straw	Late fattening steer	1	609.0	6.6	13.22	3.28	28.39	16.20	4.30	3.33	3.05	0.9136	4.2	4.1	HHCS
[3]	Corn + rice straw	Late fattening steer	1	598.0	6.7	13.22	3.28	28.39	16.20	4.33	3.28	2.96	0.9000	6.0	4.1	HHCS
[3]	Corn + rice straw	Late fattening steer	1	603.0	6.7	13.22	3.28	28.39	16.20	4.33	3.45	3.06	0.8874	5.9	5.2	HHCS
[3]	Barley + rice straw	Late fattening steer	1	647.0	7.9	12.42	1.97	34.56	22.14	4.25	3.24	2.99	0.9219	5.7	2.3	HHCS
[3]	Barley + rice straw	Late fattening steer	1	595.0	7.8	12.42	1.97	34.56	22.14	4.26	3.13	2.83	0.9057	5.8	3.3	HHCS
[3]	Barley + rice straw	Late fattening steer	1	579.0	7.8	12.42	1.97	34.56	22.14	4.23	3.26	2.96	0.9094	6.2	3.1	HHCS
[3]	Barley + rice straw	Late fattening steer	1	614.0	7.8	12.42	1.97	34.56	22.14	4.24	3.22	2.91	0.9044	6.4	3.2	HHCS
[3]	Barley + rice straw	Late fattening steer	1	639.0	7.7	12.42	1.97	34.56	22.14	4.23	3.36	3.03	0.8996	5.6	4.6	HHCS
[3]	Corn + rice straw	Late fattening steer	1	607.0	7.5	13.22	3.28	28.39	16.20	4.32	3.39	3.12	0.9213	5.7	2.0	HHCS
[3]	Corn + rice straw	Late fattening steer	1	589.0	7.6	13.22	3.28	28.39	16.20	4.29	3.25	2.87	0.8826	7.4	4.5	HHCS

[3]	Corn + rice straw	Late fattening steer	1	611.0	7.5	13.22	3.28	28.39	16.20	4.32	3.32	2.89	0.8715	9.4	3.6	HHCS
[3]	Corn + rice straw	Late fattening steer	1	650.0	7.8	13.22	3.28	28.39	16.20	4.28	3.51	3.15	0.8978	5.8	4.4	HHCS
[3]	Corn + rice straw	Late fattening steer	1	588.0	7.7	13.22	3.28	28.39	16.20	4.27	3.14	2.82	0.8967	6.6	3.7	HHCS
[3]	Corn + rice straw	Late fattening steer	1	649.0	7.8	13.22	3.28	28.39	16.20	4.28	3.29	2.81	0.8521	8.9	6.2	HHCS
[4]	Rice straw + Concentrate	Growing heifers	1	159.0	2.4	10.46	2.43	48.19	27.66	4.13	3.00	2.50	0.8333	10.4	6.9	HHCS
[4]	Rice straw + Concentrate	Growing heifers	1	149.0	2.4	10.46	2.43	48.19	27.66	4.08	3.04	2.50	0.8219	9.1	8.2	HHCS
[4]	Rice straw + Concentrate	Growing heifers	1	149.0	3.4	10.46	2.43	48.19	27.66	4.24	3.00	2.56	0.8529	10.3	3.9	HHCS
[4]	Rice straw + Concentrate	Growing heifers	1	146.0	3.5	10.46	2.43	48.19	27.66	4.23	2.89	2.49	0.8614	10.1	4.0	HHCS
[4]	Rice straw + Concentrate	Growing heifers	1	177.0	3.9	10.46	2.43	48.19	27.66	4.33	3.26	2.85	0.8740	7.9	4.7	HHCS
[4]	Rice straw + Concentrate	Growing heifers	1	162.0	3.7	10.46	2.43	48.19	27.66	4.30	3.41	3.00	0.8810	7.5	4.8	HHCS
[4]	Rice straw + Concentrate	Heifers	1	227.0	2.6	9.86	2.19	48.47	27.16	4.35	3.31	2.69	0.8140	11.1	8.1	HHCS
[4]	Rice straw + Concentrate	Heifers	1	213.0	2.6	9.86	2.19	48.47	27.16	4.35	3.27	2.62	0.8000	11.9	8.2	HHCS
[4]	Rice straw + Concentrate	Heifers	1	198.0	2.6	9.86	2.19	48.47	27.16	4.35	3.27	2.58	0.7882	11.6	8.2	HHCS
[4]	Rice straw + Concentrate	Heifers	1	213.0	3.8	9.89	2.19	48.30	27.00	4.26	3.21	2.61	0.8115	10.6	9.0	HHCS
[4]	Rice straw + Concentrate	Heifers	1	209.0	3.8	9.89	2.19	48.30	27.00	4.26	3.21	2.71	0.8443	8.0	7.4	HHCS
[4]	Rice straw + Concentrate	Heifers	1	194.0	3.8	9.89	2.19	48.30	27.00	4.26	3.16	2.53	0.8000	9.8	10.8	HHCS
[4]	Rice straw + Concentrate	Heifers	1	197.0	3.8	9.89	2.19	48.30	27.00	4.26	3.05	2.53	0.8276	8.8	8.6	HHCS
[4]	Rice straw + Concentrate	Heifers	1	224.0	3.8	9.89	2.19	48.30	27.00	4.26	3.08	2.42	0.7863	14.5	6.8	HHCS

[4]	Rice straw + Concentrate	Heifers	1	228.0	4.7	9.95	2.20	48.00	26.73	4.30	3.09	2.47	0.8000	10.4	9.7	HHCS
[4]	Rice straw + Concentrate	Heifers	1	206.0	4.7	9.95	2.20	48.00	26.73	4.30	3.11	2.60	0.8356	7.7	8.2	HHCS
[4]	Rice straw + Concentrate	Heifers	1	206.0	4.7	9.95	2.20	48.00	26.73	4.30	3.38	2.81	0.8302	10.4	6.3	HHCS
[4]	Rice straw + Concentrate	Heifers	1	201.0	4.7	9.95	2.20	48.00	26.73	4.30	3.40	2.70	0.7938	9.3	11.3	HHCS
[4]	Rice straw + Concentrate	Heifers	1	181.0	4.7	9.95	2.20	48.00	26.73	4.28	3.13	2.57	0.8231	8.3	9.5	HHCS
[4]	Rice straw + Concentrate	Heifers	1	220.0	4.7	9.95	2.20	48.00	26.73	4.30	3.13	2.60	0.8299	10.0	6.8	HHCS
[4]	Rice straw + Concentrate	Steers	1	275.5	5.5	12.66	1.84	55.46	29.33	3.76	2.22	2.04	0.9180	6.6	1.4	HHCS
[4]	Rice straw + Concentrate	Steers	1	239.5	5.0	12.65	1.84	55.49	29.36	3.76	2.14	1.98	0.9252	6.5	0.6	HHCS
[4]	Rice straw + Concentrate	Steers	1	222.0	4.4	12.64	1.84	55.51	29.39	3.77	2.09	1.93	0.9239	7.6	0.2	HHCS
[4]	Rice straw + Concentrate	Steers	1	222.0	4.5	12.64	1.84	55.51	29.39	3.76	2.09	1.93	0.9255	7.4	0.2	HHCS
[4]	Rice straw + Concentrate	Steers	1	206.5	4.3	12.66	1.84	55.48	29.35	3.74	2.07	1.93	0.9326	6.7	0.3	HHCS
[4]	Rice straw + Concentrate	Steers	1	179.0	3.9	12.64	1.84	55.51	29.39	3.72	1.97	1.82	0.9221	7.8	0.1	HHCS
[4]	Rice straw + Concentrate	Steers	1	246.5	4.0	14.13	1.93	52.87	26.23	3.75	2.28	2.13	0.9341	6.6	0.8	HHCS
[4]	Rice straw + Concentrate	Steers	1	224.5	3.7	12.64	1.84	55.50	29.38	3.70	2.14	1.97	0.9241	7.6	0.8	HHCS
[4]	Rice straw + Concentrate	Steers	1	215.5	3.4	12.65	1.84	55.49	29.37	3.74	2.12	1.94	0.9167	6.9	0.4	HHCS
[4]	Rice straw + Concentrate	Steers	1	197.5	3.2	12.65	1.84	55.49	29.36	3.81	2.09	1.94	0.9254	7.5	0.9	HHCS

[4]	Rice straw + Concentrate	Steers	1	196.5	3.2	12.65	1.84	55.50	29.38	3.78	2.09	1.94	0.9254	7.5	0.0	HHCS
[4]	Rice straw + Concentrate	Steers	1	163.5	2.9	12.64	1.84	55.50	29.38	3.79	2.28	2.10	0.9242	6.1	0.5	HHCS
[4]	Rice straw + Concentrate	Steers	1	227.5	2.7	12.60	1.83	55.57	29.47	3.70	2.26	2.07	0.9180	6.6	0.2	HHCS
[4]	Rice straw + Concentrate	Steers	1	207.0	2.5	12.60	1.83	55.58	29.47	3.72	2.28	2.12	0.9298	7.0	0.2	HHCS
[4]	Rice straw + Concentrate	Steers	1	183.0	2.1	12.60	1.83	55.57	29.46	3.76	2.10	1.90	0.9091	9.1	0.6	HHCS
[4]	Rice straw + Concentrate	Steers	1	166.5	2.0	12.60	1.83	55.59	29.48	3.70	2.10	1.90	0.9048	9.5	0.3	HHCS
[4]	Rice straw + Concentrate	Steers	1	187.5	2.3	12.65	1.84	55.48	29.36	3.74	2.04	1.87	0.9149	8.5	0.0	HHCS
[4]	Rice straw + Concentrate	Steers	1	149.5	2.0	12.64	1.84	55.50	29.38	3.70	2.15	2.00	0.9302	7.0	0.1	HHCS
[5]	Rice straw + Concentrate	Steers	1	383.0	7.2	12.62	1.84	55.54	29.42	3.88	2.25	2.04	0.9074	8.0	1.2	HHCS
[5]	Rice straw + Concentrate	Steers	1	359.0	7.4	12.62	1.84	55.54	29.42	4.00	2.41	2.19	0.9101	7.3	2.2	HHCS
[5]	Rice straw + Concentrate	Steers	1	396.5	5.6	12.60	1.83	55.59	29.48	3.89	2.23	1.91	0.8560	12.8	0.8	HHCS
[5]	Rice straw + Concentrate	Steers	1	384.5	5.8	12.60	1.83	55.59	29.48	3.98	2.41	2.10	0.8714	11.4	0.7	HHCS
[5]	Rice straw + Concentrate	Steers	1	366.5	4.0	12.55	1.83	55.67	29.59	3.93	2.25	1.93	0.8556	13.3	1.1	HHCS
[5]	Rice straw + Concentrate	Steers	1	376.0	4.1	12.55	1.83	55.67	29.59	4.05	2.41	2.02	0.8384	13.1	4.0	HHCS

[1]HHCS, head hood chamber system; ADF, acid detergent fibre; CP, crude protein; DE, digestible energy; EE, ether extract; GE, gross energy; ME, metabolisable energy; NDF, neutral detergent fibre.

References

1. Thak, T.Y.; Kang, T.H.; Kim, K.S. Studies on maintenance requirements of energy and protein for Korean native cows by metabolism trials. Korean J. Anim. Sci. 1**983**.
2. Seol, Y.-J.; Kim, K.-H.; Baek, Y.-C.; Lee, S.-C.; Ok, J.-W.; Lee, K.-Y.; Hong, S.-K.; Park, K.-H.; Choi, C.-W.; Lee, S.-S. Comparison of methane production in Korean native cattle (Hanwoo) fed different grain sources. J. Anim. Sci. Technol. **2011**, 53, 161–169.
3. Seol, Y.-J.; Kim, K.-H.; Baek, Y.-C.; Lee, S.-C.; Ok, J.-W.; Lee, K.-Y.; Choi, C.-W.; Lee, S.-S.; Oh, Y.-K. Effect of Grain Sources on the Ruminal Methane Production in Hanwoo Steers. J. Anim. Sci. Technol. **2012**, 54, 15–22.
4. RDA Determination of methane emission factors from enteric fermentation of large ruminant animals: in the final report on quantifying greenhouse gas emissions and developing emission factors in livestocl agriculture sector (Project number PJ006785 in Korean).; Rural Development Administration, Korea, **2014**.
5. Kim, K.H.; Oh, Y.G.; Kim, W.; Lee, S.C.; Shin, K.J.; Jeon, B.T. determination of energy requirements for maintenance in Hanwoo steers. J. Anim. Sci. Technol. **2004**, 46, 193–200.
